# Experiences of Dysphagia after Stroke: An Interview Study of Stroke Survivors and Their Informal Caregivers

**DOI:** 10.3390/geriatrics4040067

**Published:** 2019-12-07

**Authors:** Sabrina A. Eltringham, Sue Pownall, Ben Bray, Craig J. Smith, Laura Piercy, Karen Sage

**Affiliations:** 1 Sheffield Teaching Hospitals NHS Foundation Trust, Speech and Language Therapy Department, SheffieldS10 2JF, UK; 2 Faculty of Health and Wellbeing, Sheffield Hallam University, Sheffield S10 2BP, UK; 3 School of Population Health and Environmental Sciences, King’s College London, London SE1 1UL, UK; 4 Division of Cardiovascular Sciences, University of Manchester, Manchester Centre for Clinical Neurosciences, Salford Royal NHS Foundation Trust, Manchester Academic Health Science Centre, Salford M6 8HD, UK; 5 The Stroke Association, London EC1V 2PR, UK

**Keywords:** acute stroke, dysphagia, patient and public involvement, thematic analysis

## Abstract

(1) Background: Swallowing difficulties (dysphagia) after stroke are not uncommon and is a consistent risk factor for stroke-associated pneumonia. This interview study explores the perspectives of stroke survivors, who had their swallowing assessed in the first few days of admission to hospital, and their informal caregivers. (2) Methods: A participatory approach was used involving people affected by stroke in the interpretation and analysis of the interview data. Data was thematically analysed and six themes were identified. (3) Results: These themes included how past-future experiences may influence a person’s emotional response to events; understanding what is happening and adjustment; the impact of dysphagia; attitudes to care; communication to patients and procedural issues. (4) Conclusion: The findings highlight the importance of effective public health messages to improve people’s responsiveness to the signs of stroke, standardisation of assessment and management procedures, effective communication to patients about the consequences of dysphagia, and the impact of dysphagia on the person who had the stroke and their informal caregiver.

## 1. Introduction

The global burden of stroke is increasing [1]. In Europe, the number of people with stroke is estimated to rise by 27% between 2017 and 2047, due to lower fatality rates and prevention strategies [2]. Stroke-associated pneumonia (SAP) is a frequent complication affecting 14% of stroke patients [3], and is associated with increased mortality [4], greater length of hospital stay and acute care costs [5], and dependency at discharge [6]. Patients are susceptible to SAP in the first days after stroke due to a combination of stroke-induced immunosuppression and material such as oral-pharyngeal secretions or gastric contents entering the lungs (aspiration) as a consequence of reduced consciousness and difficulty swallowing (dysphagia) [7]. Post-stroke dysphagia occurs in 37–78% of patients and increases risk of pneumonia ≥3-fold and 11-fold in patients with confirmed aspiration [8].

Early dysphagia screening and specialist assessment by a speech and language pathologist (SLP) is associated with reduced risk of developing SAP [9]. In the United Kingdom, guidelines [10] recommend people with a stroke are screened for dysphagia within 4 h of admission to hospital and, if dysphagia is suspected, a comprehensive swallow assessment, usually carried out by a SLP, is undertaken within 72 h. A wide range of dysphagia screening protocols (DSPs) are used to screen people and there is limited information about what comprises a specialist swallow assessment [11]. A range of medical interventions and clinical processes may also be associated with risk of SAP in people with swallowing difficulties [12].

### Aims of the Study

This study forms part of a series of studies [11,12,13] that aim to investigate how variation in assessment and management of dysphagia in acute stroke affects development of SAP. The aim of this interview study is to explore the experiences of people with swallowing difficulties following a stroke to give a more rounded picture of delivery of care and ensure that the perspectives of stroke survivors and their informal caregivers are included. We wanted to include the patient story of swallowing difficulties post-stroke as a way to better understand the patient experience of dysphagia assessment and management, as well as the views of the staff involved [13,14]. Including the patient story has the potential to highlight variations in practice from the perspective of the persons affected, thereby providing a more inclusive understanding of service delivery. Informal caregivers can also provide another dimension and a contribution to better understanding of patient care as well as insight into their role. We wanted to extend beyond formal data collection against narrow performance indicators by interviewing people who had a swallow assessment during the first 72 h of admission into hospital, alongside other clinical processes and/or medical interventions that take place in the first few days post-stroke.

The study also wanted to actively involve stroke survivors not as research subjects but as research partners within the research process to help analyse the interview data and build the themes collaboratively with the academic research team. We wanted to involve people affected by stroke because of the unique insights they can bring thereby helping to ensure the relevance and quality of the research but also because it is a core democratic principle that people affected by research have a right to have a say in how publicly funded research is undertaken [15].

## 2. Methods

### 2.1. Qualitative Approach and Research Paradigm

The interviews reflect the ontological assumption that reality is shaped by experience and the epistemological perspective that a subjective representation of this reality is being presented from the researchers’ perception. Interviews were chosen for their ability to provide a rich source of information about people’s experiences of having their swallowing assessed during the acute phase of stroke and their opinions and feelings associated with these experiences. A participatory methods approach involving a group of people affected by stroke in the data analysis embodies a process by which the analysis and interpretation of the data is not the sole responsibility of the researcher but a shared responsibility with the people themselves.

### 2.2. Researcher Characteristics and Reflexivity

The primary author is a SLP working in an acute hospital. There was the possibility that she may have had contact with the sample population whilst working in a clinical role. To avoid the risk of any researcher-practitioner conflict, the researcher did not recruit a patient or informal caregiver with whom she had any direct clinical contact. During an interview, there was also the potential for a participant to disclose something, which could present harm either to themselves or others. Participants were informed of the boundaries of confidentiality and as such, what could not be held as confidential [16]. Conducting the interviews meant that the researcher had some prior knowledge of the data and some initial analytic thoughts. The primary author kept a reflective log of initial interpretations and sought to challenge any assumptions by embracing alternative or counter information.

### 2.3. Environment

The context for the interviews was dependent on where the person was in the stroke pathway and their preferred setting. This included the acute stroke unit, the stroke rehabilitation unit or the person’s home (Supplementary Material—Table S1). Participants were asked if they would like their informal caregiver to attend the interview to support them during the interview.

### 2.4. Sampling Strategy

Participants were identified from a convenience sample from a case note review from a single hospital. The sample included patients who had a dysphagia screen on admission and who went on to have a swallow assessment by a SLP or an equivalent trained professional. The sample size for the interviews was based on the objectives of the patient interviews in context of the overall research project [17] and feedback from service users.

### 2.5. Ethical Approval

The research obtained ethics approval from London-Bromley Research Ethics Committee (REC Ref 18/LO/0096) and the primary authors’ academic institution REC (Ethic Review ID ER5599201). Potential participants were approached and provided with information about the study. Those who agreed to participate were invited to an interview and written consent obtained before participation.

### 2.6. Data Collection Method and Instruments

Interviews were conducted between 17 April 2018–12 June 2018 by the primary author and digitally recorded using an Olympus WS-853 digital voice recorder.

A topic guide was developed in response to a direct request from the patient and public involvement (PPI) group involved in the research study that service user perspectives be included. The guidelines for dysphagia screening and assessment in acute stroke care [10] were used to help frame and sequence questions about what happened to an individual in the first few days post-stroke. The guide was exploratory and open questions were used, in order to elicit spontaneous descriptions of respondents’ experiences. Visual materials such as calendars were used to help people recall when they had the stroke and the first few days following.

### 2.7. Units of Study

Face-to-face interviews were conducted with five people with stroke. The person with the stroke was the sole participant in three interviews and two involved the person and their informal caregiver. There was one informal caregiver only interview (Supplementary Material—Table S1). The time of the interview ranged between 8 and 100 days post-stroke onset.

### 2.8. Data Processing

The digital recording of each interview was uploaded to the primary author’s academic institution Research Store and deleted from the recording device. The primary author transcribed each audio file and any potential sources of identification were made anonymous.

### 2.9. Data Analysis

There were three stages to the data analysis. The first stage involved the researcher and the academic research team. This began with the primary author familiarising herself with the interview data. The transcripts were read and reread and segments of text were identified, coded manually and categorised into themes. As part of the iterative process, the academic research team also reviewed the themes.

The second stage of the analysis involved three members of the Stroke Association’s ‘Stroke Voices in Research’ (SVR) panel. Members who had previously expressed an interest in being involved in research about swallowing were sent information about the study and scope of the involvement and invited to attend a half-day focus group. Steps were taken to allow members to engage meaningfully in the analysis. This included tailoring support and resources to individual needs. Anonymised transcripts in audio and written formats and a glossary of terms were provided in advance of the meeting, and members were able to contact the primary author if they had any questions during the course of their involvement. At the focus group, the researcher facilitated members to discuss their interpretations of the interviews and identify segments of data as evidence. Together, they explored if any of the points raised cut across more than one interview and labelled and categorised the data. In addition, the group reviewed and validated the themes developed by the main author, independently identifying and labelling the same segments of data.

The final stage of the process was to triangulate the findings from the focus group with the research team analyses and define and name the themes.

### 2.10. Techniques to Enhance Trustworthiness

Several techniques were used to ensure the trustworthiness and credibility of the data analysis. During the interview, the primary author asked probing and interpreting questions [18] to pursue an answer and to clarify what was said. The SVR panel and the research team provided peer validation of the interview themes. The Standards for Reporting Qualitative Research (SRQR) [19] was used for transparency of reporting. The Guidance for Reporting Involvement of Patients and the Public (GRIPP2) [20] was used for the reporting of patient and public involvement (PPI) in the research.

## 3. Results

Six themes were identified. These included (1). Past-future experiences; (2). Understanding what is happening and adjustment to the stroke; (3). Impact of dysphagia; (4). Attitudes to care; (5). Communication by staff to the individuals affected by stroke; and (6). Procedural issues. The first four themes were developed by the researcher and validated by the SVR panel members. Where the SVR Panel enriched these themes is identified. The fifth and sixth themes were new themes identified by the panel members and developed during the focus group. The themes are elaborated using quotations from original data to substantiate the analytic findings.

The results also include matters identified by the SVR panel that the participants did not raise and which were relevant to their own experiences. This section is entitled ‘The Unsaid’.

### 3.1. Past-Future Experiences

This theme describes how people’s past-future experiences may influence a person’s emotional response or understanding of procedures, medical interventions and concerns about risk of developing pneumonia. One participant had a choking episode, which resulted in a cardiac arrest call. Since his stroke, his informal caregiver had become aware of the recommended guidelines for people to remain nil by mouth (NBM) until screened for dysphagia. This new knowledge led her to reflect on what should have happened and the potential consequences;

*“What I couldn’t understand was is in all the leaflets I’ve got there it says they test you for your swallowing when you first go in, but you were never tested, were you?”*

*(P35)*

*“Luckily he survived but if that domestic hadn’t been there, who knows.”*

*(P35)*

The same person required a percutaneous endoscopic gastrostomy (PEG), a procedure in which a flexible feeding tube is placed into the stomach, which allows nutrition and fluids to be put directly into the stomach bypassing the mouth and oesophagus. His caregiver’s emotional response to this news had been shaped by their friends’ experiences;

*“Then they said he’d have to have the PEG (Percutaneous endoscopic gastrostomy), which was a bit upsetting for us because we knew three people who were PEG-fed and he’s always said must be terrible to have to be fed like that and then it happened to him.”*

*(P35)*

One participant’s parents had both died of pneumonia and his expectation of developing pneumonia was influenced by his parents’ experience. He was *“expecting repercussions” (P38)* as a consequence of his dysphagia. His informal caregiver stated, *“He was convinced”* he was going to develop pneumonia. One member of the SVR panel perceived that the person’s concerns may have been a consequence of whoever had explained the risk of pneumonia to the participant, and that they “may have overdone it”. The Panel member perceived the person to be still concerned about developing a chest infection.

### 3.2. Understanding What Is Happening and Adjustment

This theme refers to a person’s understanding of what is happening at the onset of their symptoms, the adjustment to the effects of the stroke and the relationship with their informal caregiver. At the start of their symptoms, two participants were unsure about what was happening to them and described feelings of *“denial” (P38, P151)*. Gradually, there was a realisation and acceptance that they were having a stroke. For one participant this was by listening to the use of the term ‘stroke’ by the ambulance crew on the way to the hospital;

“*This is a stroke ambulance and we go to [hospital name].” ”OK, stroke, we go the [hospital name] right.”*

*(P38)*

For the other participant when he realised he couldn’t swallow, he left the dinner table and went out into the garden where he became more self-aware as his symptoms evolved;

*“I was panicking inside and then I started to feel my right side tickling, my right face here my arm my leg. Then I knew in my own heart what it were. I knew I were having some kind of stroke.”*

*(P151)*

Members of the SVR panel likened these responses of ‘denial’ and ‘acceptance’ to the five stages of dying based on the works of Elizabeth Kubler-Ross [21], whose model has been applied to many stages of grief and loss. The panel perceived another participant displayed feelings of anger, which is another stage of grief, about his wife’s rehabilitation. The panel felt the participant’s emotional response was a consequence of unrealistic expectations of his wife’s exercise tolerance, which was attributed to a lack of understanding due to poor communication by staff (see also Section 3.5—Communication to Patients).

Other examples of participants processing what had happened related to why recommended procedures had not been followed, the components of the SLP assessment and the rationale for the SLP swallowing recommendations.

*“He’s never actually had it tested when he first got in to see if he could swallow … I mean, at first, I didn’t think anything about it, but reading all the bumf I’ve got I thought, well, he should have been tested for this straight away.”*

*(P35)*

*“But what surprises me was the first time they tried him with anything by mouth was sips of orange juice off the spoon and yet he can’t have any liquids now.”*

*(P35)*

It was the psychological effects of the stroke, which surprised another informal caregiver; *“What surprised me … is the mental effect it has on patients … because the mind seems to go and wander, could be over there somewhere or up there somewhere.”* He spoke about the cognitive effect of the stroke on the person involved (P89), the adjustment and repair and how they were adapting to living with the long-term effects of stroke with the installation of equipment to help with daily living, when the participant returned home;

*“We’ve got furniture in the house and a sling in, even though I don’t like them.”*

*(P89)*

The SVR Panel identified the participant’s expression. *“I’m frightened of them”, (P89)* when she was talking about the portable hoist equipment.

One participant *(P133)* described adjusting to the physical effects of the stroke. This included living with dizziness, feeling *“dry all the time”* and changes to her voice. The person felt *“frightened”* to go out, and described her voice like a *“darlek”*, which made her *“feel a bit down”*. One SVR member empathised that she could not drink tea anymore; *“I used to love a cup of tea and that but then I can’t face one”.* Two participants used the term *“dark” (P89, P151)* to describe the impact of their stroke but had different attitudes. One participant stated, *“I call it my dark place where I don’t like and don’t want to be but I can’t do anything about it” (P89)*. The other stated, *“It’s a dark time when you’re laid there and your family’s here and your family’s upset and you think ‘Why me?’ but then you look around and you see other people that’s a lot worse than you and it’s a wake-up call, that to say ‘Stop feeling sorry for yourself’” (P151).* One member of the SVR panel associated the participant’s *“dark time”* with being unable to eat. For two participants, as they regained their independence, they believed that their determination was the key to their recovery: *“I was determined I was going to walk I wasn’t going to be messed about” (P155).*

Despite living with the ongoing effects of stroke, two participants described feeling they were lucky; *“I’ve been one of the lucky ones” (P151)* or their partner was lucky; *“So he was very lucky really and I think he was also lucky the fact that he didn’t lose his speech and upper body. I mean he can use his hands, so he wasn’t affected that way, but was just his walking and his swallow” (P35).* One SVR panel member perceived this positive outlook to be a consequence of people measuring themselves against others, which resonated with his experience of a high rate of mortality of fellow patients in the first 48 h after his stroke.

### 3.3. Impact of Dysphagia

This theme describes the impact of having a swallowing difficulty on participating in social activities, participants’ reactions to modified food and thickened drinks, and their swallowing ability and having a nasogastric tube (NGT) inserted.

One informal caregiver spoke about the impact of having a PEG on being able to continue to socialise and participate in family celebrations, going out, and the loss of the enjoyment of eating;

*“I thought, it’s cruel really because what’s happened to him because he really loves food and it’s part of your social life: going out having a meal, having people to the house and going out with friends and family, isn’t it?”.*

*(P35)*

Members of the SVR panel also noted the importance of the stroke survivor going to the dining room for their meals. One participant’s *(P89)* caregiver stated, *“I think is a good thing about being here, they take her to the dining room every meal”.* Two SVR members felt differently about the caregiver leaving at mealtimes rather than waiting for his wife to return from the dining room. One member perceived his behaviour as “a bit harsh”, while the other member felt he was helping with his wife’s rehabilitation. As well as the social participation of dining with others, the panel also identified participants’ embarrassment of not being able to eat independently and eating in front of others.

Two participants had strong emotional reactions to being recommended thickened fluids and modified food by the SLP. They referred to the thickened drinks as *“horrible” (P133, P155)* and like *“sludge” (P133),* and described the modified diet as *“slop” (P155)*. The use of these emotive terms contrasted sharply with professional terminology used by the researcher, for example, pureed diet. To avoid drinking the thickened drinks, a family member would buy thick yoghurts as an alternative. Members of the SVR panel identified how the informal caregiver had bought more palatable alternatives. Based on their interpretation of the transcripts, they questioned if patients and caregivers were given adequate written advice on suitable foods and the importance of maintaining a balanced nutritional diet. The SVR panel also perceived a “casualness” with regards to the thickening of patient’s drinks and queried if staff responsible for thickening drinks were following the recommended guidelines. One participant stated the drink *“used to make it right thick” (P133)* and another, *“I don’t know how much they had to put in, perhaps a spoonful of powder or something” (P155).* Both participants demonstrated an understanding of what consistency and types of foods they were able to manage or should avoid; *“I can’t eat anything with a crust on or anything like that, or else it gets stuck in my throat” (P133)* and *“I’m not on normal food now, but I’m on mashed-up food and … I can drink anything now” (P155).* The perception of being able to eat *“normal”* food was associated with emotional and dysphagia recovery. Participants had a pragmatic response to their treatment and swallow recovery: *“There’s a tunnel there and I’m getting to the end of it. Nothing will stop me” (P151).*

Informal caregivers were involved with the implementation of the SLP dysphagia recommendations. This included communicating the advice to the wider family, adherence to the advice and how that made them feel, providing assistance to eat and drink, and preparation of meals. One participant’s *(P38)* caregiver acted as a conduit by updating the family on the SLP swallowing recommendations; *“I was keeping my family orientated ‘cos all my family lives away”*. She described how she felt *“cruel”* for not giving her husband more to drink when he was on strict swallowing trials. The caregiver appeared to attribute responsibility for this to herself rather than to external factors such as professional advice: *“We sounded very cruel ‘cos he kept saying, ‘Can I have a drink?’ … No, you can’t you’ve had your five sips of water”.* This was made more difficult because her husband could not see any reason why he could not have any more.

Informal caregivers facilitated the implementation of the recommendations by assisting with mealtimes; *“And I used to feed you” (P89)*, and preparing meals *“I made that [rhubarb and custard fool] for him” (P35)*, which adhered to the SLP consistencies. Members of the SVR panel highlighted the potential burden on caregivers when their loved one returned home. Issues highlighted were the cessation of their own social activities, the additional care duties of preparing meals and assisting with PEG feeds, and the potential implications for their own emotional and physical wellbeing.

Three participants spoke about the discomfort of having the NGT inserted; *“not very comfortable at all” (P89)*, *“I don’t want that (Fibre endoscopic evaluation of swallowing), this (NGT) was bad enough” (P151)* and *“having it fitted was not very pleasant … having it there at times was quite painful because it would catch on different things” (P38)*. One SVR member who had experience of NGT insertion empathised with the discomfort. One caregiver felt the displacement and reinsertion of the NGT and confirmation of positioning had likely lengthened the time her husband (P38) had the NGT: *“We had to have the tubes fitted several times because it dislodged … so then you have to back down to the X-ray to make sure it’s in the right place … so that’s probably lengthened the time that he would because of it.”* The importance of sustaining adequate nutrition and hydration was identified by the panel as important for the avoidance of worsening stroke symptoms.

### 3.4. Attitudes about Care

This theme refers to participants’ attitude to treatment and awareness of staff roles. Participants praised the care they received and the attentiveness of the staff. An alternative interpretation from members of the SVR panel, of the attentiveness of the staff to the participant who choked, was that staff were trying to ameliorate the situation. Despite the incident, the informal caregiver felt *“everybody was there when it was needed” (P35).* Another caregiver had mixed feelings about the care and felt *“everything that has happened has gone too slow” (P89)*. The transition of one of the hospital stroke wards to a rehabilitation unit was felt to have impacted on the level of staffing on the stroke ward. This had direct consequences for personal care, which led to his wife feeling *“embarrassed all the time”* by her incontinence. They both spoke favorably about staff that spent time assisting at lunchtime.

Members of the SVR panel perceived a lack of recall on the part of many participants about when, where and what the swallow test was. One participant was aware of having lots of tests but did not always understand who people were and the SLP was sometimes confused with the occupational therapist. The same participant described how they (the person with stroke), the staff and their family were part of a team: *“It’s a team, it gets you through it. It’s not just yourself, it’s a team. The hospital is a team. Your family is a team. When you’ve got that you’ll get through it” (P151).* An informal caregiver described the sense of relief knowing that her husband *(P38)* was being looked after *“It’s the relief of knowing that somebody’s looking after who knows what they’re doing is looking after him.”*

### 3.5. Communication to Patients

This theme evolved from the analysis and interpretation of the data by individual members of the SVR panel and their collective discussion. The theme refers to examples of good and poor communication with patients and their informal caregivers about medical interventions, the risks of developing pneumonia, information about maintaining adequate nutrition and hydration, and the impact of stroke.

A perceived example of good communication about the impact of stroke and medical interventions included one participant’s understanding of the impact of stroke on the swallowing process and the rationale for swallowing rehabilitation. The participant was having neuromuscular electrical stimulation swallowing therapy. The focus group agreed that the participant had a good understanding of his stroke and the rationale for the therapy.

An example of how communication about medical interventions was perceived as potentially lacking was the explanation to patients for the need for alternative long-term nutrition. One member of the SVR panel felt that staff should not be raising the matter of PEG with a patient until it had been established that feeding via a NGT was not going to work. A second example where communication about nutrition was felt to be potentially lacking was information about the importance of nutrition for physiological recovery and how to maintain adequate nutrition and hydration (see also Section 3.3–Impact of dysphagia). SVR members perceived there to be a lack of information about nil by mouth status or if a patient was allowed oral intake. They identified that a participant had referred to a swallowing notice being changed behind the person’s bed when a person’s SLP recommendations was updated.

Members acknowledged that the assessment and management of dysphagia was tailored to the individual but highlighted what they perceived to be a lack of standardisation. For example, SVR members felt communication about the risks of developing pneumonia may have been lacking when one participant stated, *“there must have been something wrong that they not found out and how I found out I don’t know … at that point pneumonia is a word I’ve heard” (P38).* The SVR panel identified how the patient could not recall how he knew about the risk of pneumonia or if he had been told or read about it. This variance was perceived to extend to recommendations for alternative nutrition and the process of moving from an NGT to a PEG, and nutritional advice. Members felt patients and their informal caregivers were left “looking for answers”.

SVR members felt it was also a lack of communication about how stroke can impact on a person’s exercise tolerance, which accounted for why one caregiver complained that; *“I don’t think she had enough physiotherapy” (P89)*. Members felt that the caregiver and the patient either did not recall or had not been informed about the fatigue effects of stroke.

### 3.6. Procedural Issues

This theme refers to procedural issues such as screening patients for dysphagia on admission in accordance with the national guidelines, and staff awareness of these procedures. There was an example where the procedures were not followed and one participant was offered food and drink before having their swallow screened despite their stroke being confirmed and the informal caregiver referring to signs of swallowing difficulties. Members identified that the person was not initially nursed in a stroke bed, which may have been a reason for staff lacking awareness. This was perceived as lack of awareness of the stroke guidelines and “fractured care” between hospitals. Members identified how it was important for all staff, including non-qualified staff, to be dysphagia aware and to know how to thicken fluids to the recommended consistency. This extended to the importance of attention to detail and for staff to be aware of subtle changes in stroke patients, and how patients should be treated on a stroke ward with staff experienced in stroke.

### 3.7. The Unsaid

This section refers to questions which participants either did not respond to, or aspects of care, which were not raised that the SVR Panel identified as important and relevant to their own experience. For example, when asked about if they required assistance with cleaning their teeth, none of the participants raised this as a concern. For one panel member, lack of oral care had been a significant issue for his relative and was surprised that participants did not respond to this question. An alternative interpretation of this lack of response might be participants did not recall some events or it reflected their strength of feeling about oral care compared to other aspects of care during the acute phase. Another member identified that none of the participants raised the matter of taking their medication.

One panel member was surprised by the “lack of angriness” by participants. He perceived the participants’ gratitude to the care they received as “all-encompassing” and that participants may have been masking how they felt or were not being entirely congruent. In contrast, another member felt there were examples of “genuine gratitude” to the National Health Service and the care they had received.

## 4. Discussion

This study provides a unique perspective of stroke patients’ experiences of having their swallow screened and assessed during the first 72 h of post-stroke and subsequent days following. Six themes were identified. These included how past-future experiences may influence a person’s emotional response to events; understanding what is happening and adjustment; the impact of dysphagia; attitudes to care; communication to patients and procedural issues. The findings highlight the importance of public health messages such as the FAST (Face, Arms, Speech, Time) [22] to help people detect and improve responsiveness to the needs of people having a stroke. It highlights how, despite these public health messages, people experience difficulty understanding what is happening at the onset of their symptoms and that there can be feelings of denial, which can lead to a delay in people’s responsiveness to calling the emergency services. At an individual level, it also highlighted that some participants did not follow the public health message to telephone the emergency services, and instead went directly to the wrong hospital resulting in delayed admission to a stroke bed and receiving a specialist swallow assessment.

The inclusion of informal caregivers in the interviews provided a measure of validation to participants’ responses and a caregiver perspective of events. Their participation not only highlighted the contribution informal caregivers make in supporting stroke patients in hospital, but also to patient safety by alerting staff to potential concerns [23], such as potential signs of aspiration. There were psychological consequences of their contribution characterised by feeling guilty after an adverse event and feeling cruel for implementing the SLP swallowing recommendations. Beyond the hospital environment, informal caregivers participated in dysphagia treatment by preparing foods to the recommended consistency and assisting with PEG feeding. The impact of living with a PEG had consequences for independence and social participation for the caregiver and the stroke survivor. The CONOCES study [24] highlighted the hidden cost of informal care and identified five indicators that predict the heavy burden borne by caregivers of stroke survivors and the likelihood of risk of burn out. These indicators were the number of caregiving hours; the patient’s health-related quality of life; the severity of stroke measured at discharge; the patient having atrial fibrillation; and the degree of dependence.

In addition to the impact of living with a PEG, participants reacted strongly to being prescribed thickened fluids and recommended modified diets. Bolus modification is often associated with worse quality of life [25]. This study highlighted the potential nutritional and hydration impact of patients avoiding meals or drinks due to their dislike of the options offered and perceived lack of understanding regarding the importance of maintaining sufficient nutritional status as part of their stroke recovery. Participants who had an NGT communicated the pain and discomfort this caused. One informal caregiver felt that the dislodgment of the NGT and subsequent confirmation of its position by chest X-ray led to the prolonged time the NGT was needed. Throughout the time the NGT was disconnected, the patient would not have been receiving the nutrition they needed.

Effective patient communication in a format that is accessible is critical in healthcare. This is even more crucial for people affected by stroke who may experience aphasia as a consequence of stroke [26]. During the first 72 h post-stroke, patients have a multitude of tests and scans and are assessed by a range of professionals. Participants were aware of having these tests but struggled to recall what they were and when they were told about the risks associated with dysphagia and developing stroke-associated pneumonia. Poor communication can lead to compromises in patient safety and dissatisfaction in patients and caregivers [27]. This was validated by the ‘Stroke Voices in Research’ panel. Other potential consequences of poor communication in this population is the risk of dehydration and malnutrition due to patients not understanding what foods and drink are suitable according to their dysphagia recommendations. The study also highlighted how a person’s past experiences have the potential to impact on their emotional response and understanding of their condition and treatment options. Time spent by clinicians finding out a person’s case history can help inform and guide shared decision-making.

Patient and public involvement in health research is a well-established principle, meaning research is carried out ‘with’ or ‘by’ members of the public rather than ‘to’, ‘about’ or ‘for’ them [15]. Compared to advising on research questions and research design, the analysis and interpretation of research data is one of the less well-explored aspects of service user involvement in research [28]. The SVR Panel helped enrich the themes identified by the researcher, checked the validity of the conclusions from a stroke survivors perspective and identified findings that were relevant to people affected by stroke, which the academic research team may have missed. The SVR identified the frequency of feeling words used by the participants to describe their emotional reactions to the stroke event and how they felt about the medical interventions and care processes. Panel members particularly empathised with the lack of communication to patients about the importance of maintaining adequate nutrition and hydration in accordance with the SLP recommendations, and they perceived a lack of standardisation in procedural issues and communication of the risk of pneumonia and the transition from short term to long term nutrition.

The National Standards for Public Involvement [29] provide a framework for reflecting on and improving the purpose, quality and consistency of public involvement in research. These standards were used to reflect on what went well and how involving service users in data analysis could be improved for the future. Standard 1: inclusive opportunities, means offering public involvement opportunities that are accessible so that research is informed by a diversity of public experience so that it leads to treatment and services that reflect the needs of the service users. Examples of what went well included working closely with the SVR Panel Coordinator and sending information about the involvement opportunity to interested and relevant members of the SVR database, with a short description of what members could expect as part of the information they received. We identified and addressed barriers to members taking part. For example, members had the option to request ‘book ahead’ transport so they did not bear any upfront costs and we made information available in different formats (Standard 4: Communications) so that it was accessible for needs of different people. We recognised that reading the transcripts might trigger feelings or emotions of the members about their own stroke experiences and offered emotional support (Standard 3: Support and Learning). For example, members were asked to be mindful about what they chose to share and knew that they could take a break from the focus group discussion and were shown a relaxation and rest room nearby where they could go if they wanted to spend some time alone or needed some one-to-one time with a member of the research team.

Involving service users in the analysis and interpretation of the data could have been further improved. Sometimes members brought up things from their own experiences, which had not always been raised by the interview participants. As part of the development of the group’s research skills, we used the group exercise to look for example quotes within the interview transcripts and explained how we had to be careful as researchers not to impose our experiences on what the participants had said and this was part of being a reflexive researcher. A learning outcome for the future would be to include more time for building research skills and discussion. This could be achieved by building on what we have learnt from this experience and actively learning from others who have involved members of the public in this stage of the research process, discuss support and training needs with new public contributors and involving public members in designing and delivering support and learning activities.

The authors acknowledge the potential limitations of the study. Firstly, the sample size is limited and reflects the opinions of a small group of stroke survivors and their informal caregivers. Secondly, the interviews took place in different settings and at different times during the stroke survivors’ pathway, which may have influenced their perspective of events in the first few days post-stroke. Thirdly, the primary author is a SLP, which may have blurred the insider-outsider boundaries of being a clinician and a researcher [30]. The main author had originally perceived that participants may struggle to recall events in the first 72 h post-stroke. This was the case with two participants. However, their informal caregivers validated what information they did provide or provided new information. There were occasions when the participant disclosed something that the researcher felt was their professional responsibility to pursue for reasons of patient safety or the interviewee sought the researcher’s professional SLP opinion. When the latter occurred, the researcher maintained boundaries and requested the participant defer their question to their own SLP. Service user involvement in the analysis and interpretation of research data also helped to check the validity of the conclusions from a public perspective.

## 5. Conclusions

This interview study has explored patient and informal caregiver experiences of patients having their swallow assessed post-acute stroke as part of a mixed-methods design study. The research has identified six themes related to this topic, including how an individual’s past-future experiences may influence their emotional response to the stroke; difficulty understanding what is happening at stroke onset and adjustment; the impact of dysphagia; attitudes to care; good and poor communication to patients; and procedural issues around screening for dysphagia. People affected by stroke were involved in analysing data and identifying themes, which were perceived as being relevant and most important to patients and their informal caregivers. The findings highlight the importance of effective public health messages to improve people’s responsiveness to the signs of stroke, standardisation of assessment and management procedures, clear and effective communication to patients about the consequences of dysphagia, and the impact of dysphagia beyond the hospital environment.

## Data Availability

Data relevant to the study are included in the article.

## Supplementary Materials

The following are available online at https://www.mdpi.com/2308-3417/4/4/67/s1. Table S1: Participant Characteristics, Table S2: Standards for Reporting Qualitative Research (SRQR), Table S3: GRIPP2 short form.
